# Model of Quality Factor for (111) 3C-SiC Double-Clamped Beams

**DOI:** 10.3390/mi16020148

**Published:** 2025-01-28

**Authors:** Angela Garofalo, Annamaria Muoio, Sergio Sapienza, Matteo Ferri, Luca Belsito, Alberto Roncaglia, Francesco La Via

**Affiliations:** 1 Physics Department, Catania University, Via S. Sofia 64, 95125 Catania, Italy; 2 Materials Science Department, Milano—Bicocca University, Via R. Cozzi 55, 20125 Milan, Italy; 3 CNR-IMM, Strada VIII, 5, 95121 Catania, Italy; annamaria.muoio@imm.cnr.it; 4 CNR-IMM Bologna Unit, Via Gobetti, 101, 40129 Bologna, Italy; sergio.sapienza@cnr.it (S.S.); matteo.ferri@cnr.it (M.F.); luca.belsito@cnr.it (L.B.); alberto.roncaglia@cnr.it (A.R.)

**Keywords:** silicon carbide, MEMS, quality factor, 3C-SiC, resonance frequency

## Abstract

Silicon carbide (SiC) is an interesting semiconductor for MEMS devices. The high-value Young’s modulus of silicon carbide facilitates high frequencies and quality (Q) factors in resonant devices built with double-clamped beams. The aim of this work is to achieve the determination and modeling of the Q-Factor for samples of micromachined 3C-SiC film on <111> silicon substrates. This study demonstrates that the experimental datasets created by Romero, integrated with the thicker samples reported in this work, fit the theoretical model presented in the paper. Furthermore, the influence of the crystallographic defects present at the 3C-SiC/Si interface on the Q-factor can be observed both in the analytical model of Romero and in the numerical model present in COMSOL. 3C-SiC layers with thickness greater than 600 nm are needed to achieve an ideal performance from double-clamped beams.

## 1. Introduction

The term MEMSs refers to micro-electromechanical systems, and it is used to describe a device or an array of devices than can range in size from a few microns to a few millimeters. This type of system constitutes a collection of microsensors which can detect and control environmental changes from the micro scale to respond on the macro scale, and obtain information about materials’ properties, geometry, and mechanical or electromagnetic effects [1]. Currently, MEMSs are everywhere; they can be found in thermal sensors, pressure sensors, resonators, mass and temperature sensors, pumps, motors, optical sensors and transmission systems [2,3,4,5,6,7,8]; in microsurgery, medical and biomedical devices [9]; and even in earthquake- [10,11,12] and volcanic eruption-monitoring sensors [13,14,15]. Given the notable success of MEMSs as sensors in the most disparate applications, it is possible to think about their eventual use in a so-called harsh environment. Thus, they must be able to operate and provide realistic results even when exposed to this type of environment for long time and should be suitable for use in a wide range of applications without sustaining damage or breakages. A harsh environment is defined as one that includes exposure to strong mechanical vibrations or sudden variations, elevated temperatures—potentially for long periods of time—highly corrosive and erosive conditions, and an intense to high level of radiation [16]. Semiconductors are materials that respond perfectly to these requests, and we have the potential to be able to exploit their properties. Silicon is the principal material in semiconductors-based MEMSs due to its good electronic and mechanical properties. The fact that silicon is the prevailing semiconductor in the industry gives it several advantages, like low-cost fabrication and is potential to be combined with other materials in the same system, compared to all others. However, silicon has limitations in application fields where performances are limited by its material properties [1]. Silicon-based systems are not the right choice for applications in harsh environments. Silicon electronics cannot operate for long periods of time at temperatures beyond 150 °C; indeed, a Si-based system is limited to electronic device efficiency below 250 °C for bulk silicon substrate devices and close to 300 °C for silicon-on-insulator (SOI) substrate devices [17,18]. Therefore, research has moved towards other alternative wide-bandgap semiconductors with good mechanical, thermal and chemical properties. Silicon carbide (SiC) is a valid alternative to replace silicon in MEMSs devices. Among SiC’s properties, its mechanical properties have been studied and analyzed so that they can be exploited for use in various applications. Such properties include its high-value Young’s modulus, which permits it to achieve higher resonance frequencies than other materials, and the quality factor (also called the Q-factor) which describes the energy loss of the system, determining the noise level in resonant sensors, which is closely related to the sensor resolution. Achieving a high-resonator-quality factor is important for applications like precision mass sensing, and, to enhance the resonator’s performance, the product of the frequency and the quality factor must reach a maximum value. The resonant frequency for micro-beam geometry depends on the residual stress of the material and the elastic properties, dimensions and geometry of the system. Varying the length and width of the resonator, it is possible to study the quality factor and resonance frequency variations. A reduction in width and an increase in length determine an increase in the quality factor due to clamping loss. Comparing several samples with different dimensions, the resonance frequency increases by 20% as the silicon carbide stress doubles, and the quality factor grows. Then, in this way, the product fxQ achieves a four-fold increase. The best value for Q is $3.41\times 10^{6}$ with a product fxQ of $9.49\times {\;10}^{11}$ Hz for an epitaxial SiC(111) on silicon substrate [19].

In this paper, we study and measure the Q-factor on double-clamped (DC) beam resonators, which is realized by micromachining of 3C-SiC films grown on (111) silicon substrates, and its maximum value as a function of the film thickness, using the analytical model described in a paper by Romero et al. [20] as a function of (111) 3C-SiC film thickness, extending the range of film thicknesses. This behavior has also been simulated numerically using COMSOL Multiphysics^®^ and a different model, and the final results are in good agreement with the analytical model.

## 2. Materials and Methods

### 2.1. Origin of Defects and Stress in 3C-SiC

Silicon carbide and silicon have similar cubic crystal structures: Si has a diamond structure with a lattice parameter equal to 0.543 nm, whereas 3C-SiC has a lattice parameter equal to 0.436 nm and has a zinc-blende crystal structure; the resulting lattice mismatch between 3C-SiC and Si is approximately 20% and contributes to the generation of compressive intrinsic stress.

The growth of epitaxial 3C-SiC layer on silicon substrate is also affected by the difference between the thermal expansion coefficients (about 8%), providing a tensile stress contribution during the final cooling step of the growth process. The lattice and thermal mismatches are the main reasons for defects in the system, typically at the interface film/substrate. The linear defects are, typically, dislocations; the planar defects are called stacking faults, while the volume defects are micro-twins (MTs), anti-phase boundaries (APBs) and protrusions. Misfit dislocation and stacking faults are the main types of detectable defects, and they are generated at the 3C-SiC/Si interface and can propagate up to the surface of the film, influencing and modifying the mechanical and electrical properties of the material. Previous works have demonstrated how the best matching at the SiC/Si interface was achieved by 5 crystalline cells of 3C-SiC, with 4 crystalline cells of Si resulting in a generation of array of misfit dislocation spaced approximately 1.6 nm from one another [21,22,23,24]. Both defects are thickness dependent. It has been observed that the density can be reduced by increasing the film thickness. The most difficult defects to remove are the stacking faults which start at the 3C-SiC/Si interface and propagate into the film. The crystal quality increases when lower growth rates are used [25]. In a recent publication by Calabretta et al. [13], it was observed that (111) 3C-SiC is extremely interesting because there is a mechanism, which is not present in (100) 3C-SiC, that produces a faster decrease in the SFs density and can have a strong effect on the performance of the MEMS devices created using this material. This later study explains the better quality of (111) 3C-SiC layers and their higher stress level that produces a resonator with higher resonance frequency and a higher Q-factor. Then, in this work, several resonators are created using this material, which is extremely promising for future MEMSs devices.

### 2.2. Sample Preparation

The 3C-SiC film growth on silicon substrates was made in a horizontal, resistively heated hot wall and low-pressure Chemical Vapor Deposition (LPCVD) system with a rotating sample holder. The heteroepitaxial growth consists of two different growth steps: first, carbonization and second, a growth ramp, with purified hydrogen ($H_{2}$) and argon (Ar) as a gas carrier, silane ($SiH_{4}$) as a silicon precursor and Propane ($C_{3}H_{8}$) as a carbon precursor.

**Figure 1:**
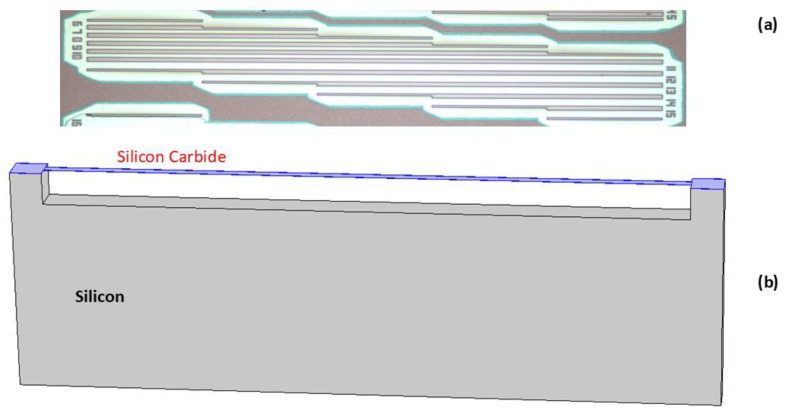
(**a**) Double-clamped beam arrays, viewed from the top, with variable length and width; (**b**) from the scheme, the two materials SiC and Si can be distinguished.

During the growth, in the reactor chamber, $N_{2}$ and tri-methyl-aluminum diluted in $H_{2}$(TMA) are introduced as N-type (N) and P-type (Al), respectively. Aluminum incorporation is realized and controlled by adding HCl to the process. The substrates are on-axis (111) bare Si with 1000 µm of thickness. All the samples are prepared with different bulk and film thicknesses, doping types (n, p), and doping levels (from NID (Non-Intentionally Doped) < $10^{16}\;cm^{-3}$, to ∼ $5\times 10^{19}\;cm^{-3}$). The fabrication process of the micromachined structures on bulk wafer is a type of front-side bulk micromachining in which part of the bulk substrate is removed by RIE etching and isotropic and anisotropic wet etching to create channels, grooves or pits in the substrate with the advantage of creating a free-standing 3C-SiC thin-film membrane, or double-clamped beams [26]. The micromachining flow is based on the following steps: by LPCVD, a 3C-SiC film is grown on silicon substrate, and a mask made of two piled layers of $SiO_{2}$ (with 1.5 µm thickness) and polycrystalline silicon (with 300 nm of thickness) are used. The next step involve lithography and self-aligned RIE etching (Reactive Ion Etching). The mask composed of polysilicon and $SiO_{2}$ is patterned and then the pattern is transferred to the 3C-SiC layer by another RIE etching step on the silicon surface. The polysilicon is eliminated, while some residual traces of $SiO_{2}$ can be found on the surface; these protect the SiC layer during the next step, when isotropic silicon plasma etching is realized. All the residual $SiO_{2}$ on the surface is removed by wet silicon oxide etching. The last step foresees additional TMAH silicon etching to increase the under-etching of the microstructures without damaging the SiC. In this way, several beams with widths of 16 μm and different lengths ranging from 200 μm to 1 mm have been realized, as reported in Figure 1. More details about the fabrication process of the resonators and the effect of the different doping methods on the Young’s modulus of the layer can be found in [27].

### 2.3. Characterization

**Figure 2:**
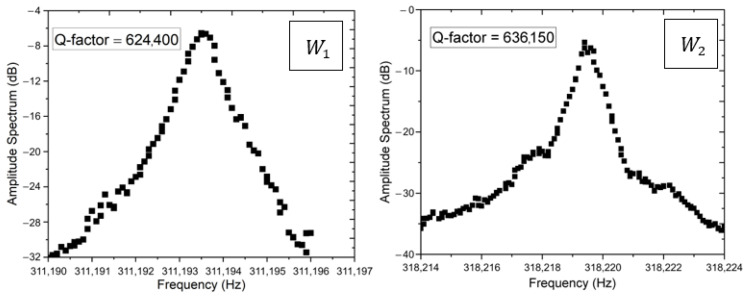
Amplitude spectrum of 1000 µm beam of W1 (**left**) and W2 (**right**). The legend shows the corresponding Q-factor value.

The measurements of the mechanical resonance frequency were performed with an optical setup based on the Doppler interferometer, in which the actuation of the resonator at different frequencies was realized using a L515A1 Thorlabs laser diode, operating at a wavelength of 515 nm in the power range 0–10 mW, connected to the output signal of an Anritsu MS2036C Network Analyzer (NA). The sensing of the induced beam vibration was carried out by connecting the output of the laser Doppler focused on the vibrating resonator to the input of the network analyzer for open-loop measurements. The measured samples were placed inside a vacuum chamber to avoid an air damping effect and the peak amplitude at the resonance frequency of beams was measured for quality factor evaluation. The Q-factor was calculated with the formula $Q=\;\frac{f_{0\;}}{∆f}$, where $f_{0\;}$ is the resonance frequency and ∆f is the Full-Width Half-Maximum (FWHM) that can be achieved with Lorentz fit (see Figure 2). The vacuum chamber was made of brass to avoid leakage and degassing and fitted with a transparent glass window to align the laser with the aid of a video camera with 10× magnification. The high vacuum in the chamber was achieved by a turbo pumping station from Pfeiffer Vacuum, below $10^{-3}$ mbar. Based on the resonance frequency, it was also possible to calculate the residual stress of the beams, which confirmed the previously results obtained in [27] with a different setup based on piezo-electric actuation.

**Figure 3:**
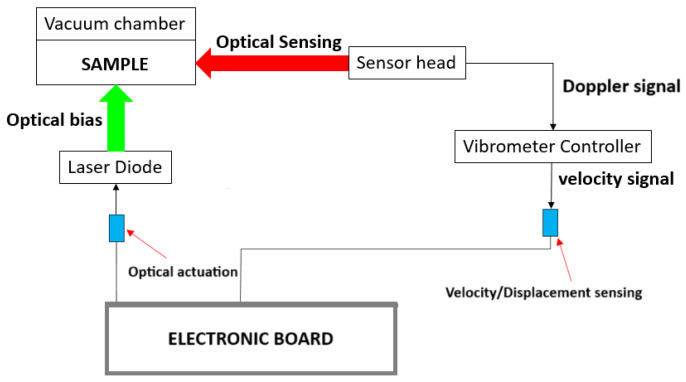
Setup for the characterization of the beam resonators.

After the first round of measurements, several results were considered inaccurate due to the frequency resolution of the NA (1 Hz) being lower than the FWHM of the peak amplitude at resonance frequency. Thus, a new setup was adopted to perform additional measurements on the same devices. In the new setup, instead of the NA, a custom electronic readout (Figure 3) was developed to produce the actuation signal controlling the green laser and to analyze the output of the Doppler vibrometer, calculating the phase and amplitude spectrum. The frequency resolution achieved with this new setup was better than the first, with a minimum frequency variation of 0.07 Hz, and the output current signal could be varied up to 46 mA for the DC value and 6 mA for the AC value.

Beam arrays with a fixed width 16 µm and length of 1000 µm, the same dimensions as the Romero samples, were measured as reported in Table 1:

## 3. Theoretical Model of Losses at the 3C-SiC/Si Interface

Some devices require a very high degree of frequency stability with respect to changes in the environment. In this application, it is important to study the sensitivity of a device’s eigenfrequencies with respect to the variation in different physical quantities, e.g., length, thickness. Simulations of the system under consideration were created below with COMSOL Multiphysics^®^ (version 6.1), a finite element analyzer, solver, and simulation software package with various applications, which is especially relevant to coupled phenomena and multiphysics. When a structure vibrates in a more complex normal mode, there are some regions of compression and some of extension, and it is associated with energy loss from the vibrational mode and corresponding damping for the resonant mode. Thermoelastic damping is particularly important in smaller MEMSs structures, in which regions of compression and expansion are in close proximity. Eigenfrequencies or natural frequencies are certain discrete frequencies at which a system is prone to vibrate. When vibrating at a certain eigenfrequency, a structure deforms into a corresponding shape: the eigenmode. An eigenfrequency analysis can only provide the shape of the mode, not the amplitude of any physical vibration.

There are several ways by which damping can be described from a mathematical point of view. The following is the equation of motion for a system with a single degree of freedom (DOF) with viscous damping and no external loads:

(1) $$m\ddot{u}+c\dot{u}+ku=0.$$

After division with the mass, m, we get a normalized form, usually written as:

(2) $$\ddot{u}+2{\zeta \omega }_{0}\dot{u}+\omega_{0}2u=0$$

Here, $\omega_{0}$ is the undamped natural frequency and ζ is called the damping ratio. For the motion to be periodic, the damping ratio must be limited to the range 0<ζ<1. The amplitude of the free vibration in this system will decay with the factor:

(3) $$e^{-\zeta \omega_{0}t}=e^{-\frac{2\pi \zeta t}{T_{0}}}$$

where $T_{0}$ is the period of the undamped vibration. The relation between the logarithmic decrement and the damping ratio is:

(4) $$\delta =\frac{2\pi \zeta }{\sqrt{1-\zeta^{2}}}\approx 2\pi \zeta$$

When a structure is subjected to a harmonic excitation at a frequency that is close to a natural frequency exactly at resonance, the vibration amplitude tends to reach infinity, unless there is some damping in the system. The actual amplitude at resonance is controlled solely by the amount of damping. In some systems, like resonators, the aim is to achieve as much amplification as possible. This leads to another popular damping measure: the quality factor or *Q* factor. It is defined as the amplification at resonance. The *Q* factor is related to the damping ratio by:

(5) $$Q=\frac{1}{2\zeta \sqrt{1-\zeta^{2}}}\approx \frac{1}{2\zeta }$$

Another starting point for the damping description is to assume that there is a certain phase shift between stress and strain. Talking about phase shifts is only meaningful for a steady-state harmonic vibration.

Given the *Q* factor and residual stress values, the aim of this work was to verify if the experimental data, combined with the experimental results in the literature on 3C-SiC (111) follow the model of quality factor described by Romero et al. [20]. The generic model for the total quality consists of five different energy-dissipation mechanisms grouped in two main categories: intrinsic and external. The external energy dissipation is due to two mechanisms: gas damping $Q_{gas}^{-1}$ and clamping loss $Q_{clamp}^{-1}$. The intrinsic dissipation consists of surface losses $Q_{surf}^{-1}$, intrinsic friction in the volume of the material $Q_{vol}^{-1}$ and thermoelastic losses $Q_{TED}$. The contribution of the thermoelastic losses has already been calculated [20] in highly stressed trampoline resonators ($Q_{TED}\sim 10^{9}$) and it is assumed to be neglected in this study due to their small contribution to thin resonators. The total quality factor is then given by:

(6) $$Q^{-1}=D^{-1}Q_{int}^{-1}+Q_{clamp}^{-1}$$

With the dilution factor D(h) depending on the dimensions (thickness h and length L), the 3C-SiC Young’s modulus E and the mean stress of the beam. Please refer to Appendix A for a thorough explanation of the Q-factor model.

One method for estimating the mean stress is to measure the resonance frequency of the released double-clamped beam. Because of the high residual stress in the structures, the resonance frequency can be simplified, depending only on the length of the beam, the mean stress and the density of the material (ρ = $3210\frac{kg}{m^{3}}\;$for SiC). For the mean stress σ as a function of the thickness of the beam, an approximated model for resonators has been developed by the following analytic expression:

(7) $$\sigma \left(h\right)=\sigma_{max}\left(1-e^{-{\left[c_{1}\left(h-h_{0}\right)\right]}^{c_{2}}}\right)$$

where the mean stress depends only on the thickness. In the analytic expression, $h_{0}$ is the transition thickness at which the mean stress transitions from compressive to tensile ($\sigma \left(h_{0}\right)=0$), $c_{1}$ is the exponential growth constant and $c_{2}$ is the kinetic order. The maximum value for the mean stress is $\sigma_{max}$.

Clamping losses are caused by phonon tunneling from the beam to the substrate through the clamping points in the form of acoustic radiation. The analytical results obtained for the contribute $Q_{clamp}$ for the double-clamped beams are reported in the Appendix.

The intrinsic dissipation $Q_{int}^{-1}$ in microresonators originates from two mechanisms: surface losses $Q_{surf}^{-1}$ and volume losses $Q_{vol}^{-1}$. The main contribution to the surface losses occurs on the top and the bottom surface of the device, as they have a larger area than the lateral surface. The volume losses are caused by the defect motion in the volume of the resonator.

When the vertical density of the defects is not uniform, as is the case for heteroepitaxial-grown 3C-SiC on Si substrate, the dissipation profile is vertically nonuniform. So, for this nonuniform vertical density of the defects, a simple model is considered for the intrinsic dissipation contribution to the quality factor. In this model, SiC film is considered as a bilayer system: the first layer is a layer with a high density of defects, with thickness $h_{0}$, near the 3C-SiC/Si interface; the second layer, with thickness $\left(h-h_{0}\right)$, is a high-quality crystal layer above the rich-defect layer, and a reduction in the defect density is expected, but total elimination is not anticipated. The contribution of the surface and volume losses to the Q-factor and all the parameters involved in the calculation are detailed in the Appendix. The parameters derived by the fitting of the experimental Q-factor data in [20] were as follows: $\beta_{hq}=\left(12\pm 5\right)\times 10^{10}\;m^{-1}$, $\beta_{def}=\left(10\pm 4\right)\times 10^{10}\;m^{-1}$ $Q_{def}^{vol}=\left(0.75\pm 0.15\right)\times 10^{3}$ and $Q_{hq}^{vol}=\left(8.0\pm 1.8\right)\times 10^{3}$. The last two parameters are related to the volumetric dissipation due to crystal dislocations, stacking faults or defects in the bulk SiC.

## 4. Comparison of Experimental Data

Given Q-factor and residual stress values, the aim of this work was to verify if our experimental data, combined with the results in the literature relating to thinner materials, are in agreement with the model of quality factor described in Romero et al.’s paper [20].

**Figure 4:**
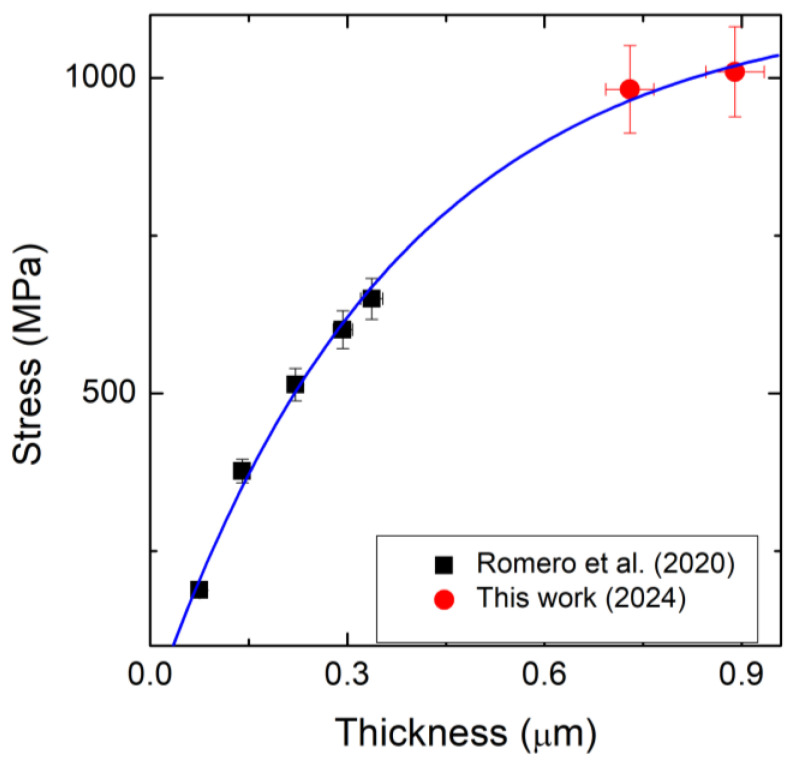
Mean stress σ(h) as a function of the thickness of the 3C-SiC film for the resonators. Black squares refer to Romero et al.’s data [20]; red circles refer to our samples.

Given the residual stress values of the beam, it was necessary to verify the trend of mean stress σ as function of the thickness h of the beam, using the analytic expression given in Equation (7). Among all the wafers used for the measurement, only two samples were acceptable for our purposes, because both the NID (Non-Intentionally Doped) and 3C-SiC film were grown on (111) silicon substrate. Therefore, combining the residual stress of our samples (red circles) with the results present in the literature for 3C-SiC films with thicknesses ranging between 75 and 340 nm (black squares) on silicon substrates [20], the total residual stress as function of the thickness of 3C-SiC films is represented in Figure 4:

According to the fit of the data using the analytical model given in Equation (3), the following calculations are achieved: maximum residual stress $\sigma_{max}$ = 1063 MPa; the transition thickness is $h_{0}=0.011\;$µm, at which the mean stress transitions from compressive to tensile ($\sigma \left(h_{0}=0\right)=0$), while the exponential growth constant $c_{1}=0.02\;nm^{-1}$ and the kinetic order $c_{2}=0.62$. For the stress fit [20], the $\sigma_{max}$ = 740 MPa, $h_{0}$ = 0.068 µm and the exponential growth constant $c_{1}=0.0015\;nm^{-1}$ and the kinetic order $c_{2}=0.54$; the different results are mainly due to the higher thickness values added in this work, which improved the precision of the fitting.

**Figure 5:**
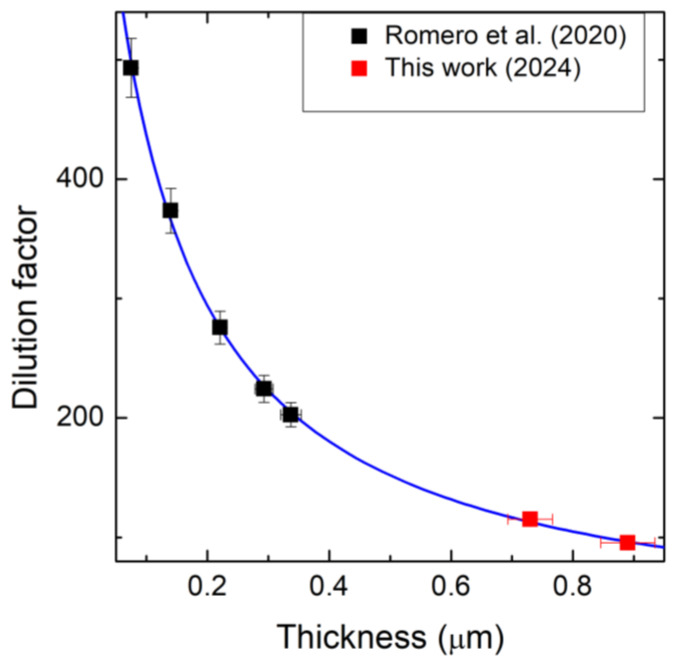
Dilution factor D(h) as function of the thickness of the 3C-SiC film for the resonators. Black squares refer to Romero et al.’s [20] data; red squares refer to our samples.

The experimental D(h) values determined by combining the measurements of the stress σ(h) and the analytical model of the thickness-dependent dilution factor for a beam of length L are represented in Figure 5.

After the determination of dilution factor D(h) values, the residual stress σ(h) and the quality factor, the intrinsic and external contributions to the total quality factor were studied by combining the data for our 3C-SiC on (111) Silicon substrates and the results in the literature [20].

**Figure 6:**
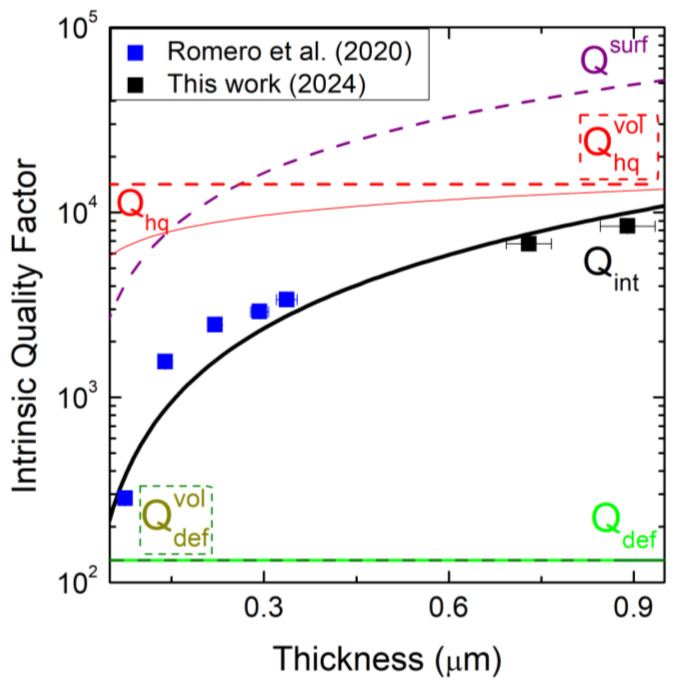
Thickness-dependent fitting intrinsic Q-factor parameters for the bilayer system. Blue squares refer to the intrinsic Q-factor values of Romero’s samples [20]; black squares refer to ours, while the black curve represents the theoretical fit. The dashed purple curve is the total surface loss curve in the bilayer system; the green curve and red curve represent the intrinsic parameter of the defect-rich and high-quality layer, respectively. Each red and green curve is limited by a dashed line, referring to the respective volume loss factor.

The experimentally determined values of $Q_{int}$, as shown in Figure 6, are deduced from Equation (1) with the analytic model for $Q_{clamp}$ and $D\left(h\right)$, while the theoretical fit is obtained using equation given by the Q-factor model maintaining $\beta_{hq}\;$and $\beta_{def}$ values [20]. For 3C-SiC films, the bilayer system is used, which involves the intrinsic parameter $Q_{def}$ of the defect-rich layer (green curve) and $Q_{hq}$ of the high-quality layer (red curve). Each layer is limited by its respective volume loss $Q_{hq}^{vol}=1.42\times 10^{4}$ and $Q_{def}^{vol}=132$, represented by a dashed red line and a dashed green line, respectively. This result of a $Q_{hq}^{vol}$ that is more than an order of magnitude of $Q_{def}^{vol}$ confirms how the rich-defect layer determines the reduction in the quality factor due to the defect at the interface. The total surface loss in the bilayer system (dashed purple line) represents the surface dissipation in both layers (high-quality and rich-defect layers).

In the intrinsic quality factor graph, as a function of the thickness of the SiC films, a result consistent with the literature was found. The same trend was expected for every intrinsic Q-factor parameter. The first difference is related to the value of $Q_{def}^{vol}=132$ and $Q_{hq}^{vol}=1.42\times 10^{5}$, in our case, and $Q_{def}^{vol}=750$ and $Q_{hq}^{vol}=8\times 10^{3}$ in the literature. The main reason for this may be due to the thickness of our samples, which is greater than the thickness in the literature, influencing the overall trends. When the thickness of the films is increased, both $Q_{def}$ and $Q_{hq}$ tend toward to the respective $Q_{def}^{vol}$ and $Q_{hq}^{vol}$ because the intrinsic dissipation in the bilayer system does not occur through surface losses at the top and bottom surface of the devices but by the defect motion in the volume. The highest value of $Q_{int}$, increasing film thickness, was found near 1 µm, but for the total quality-factor curve, the external clamping loss must be calculated to observe the eventual deviations. The last step of this work is related to the study of the total quality factor, considering several trends.

**Figure 7:**
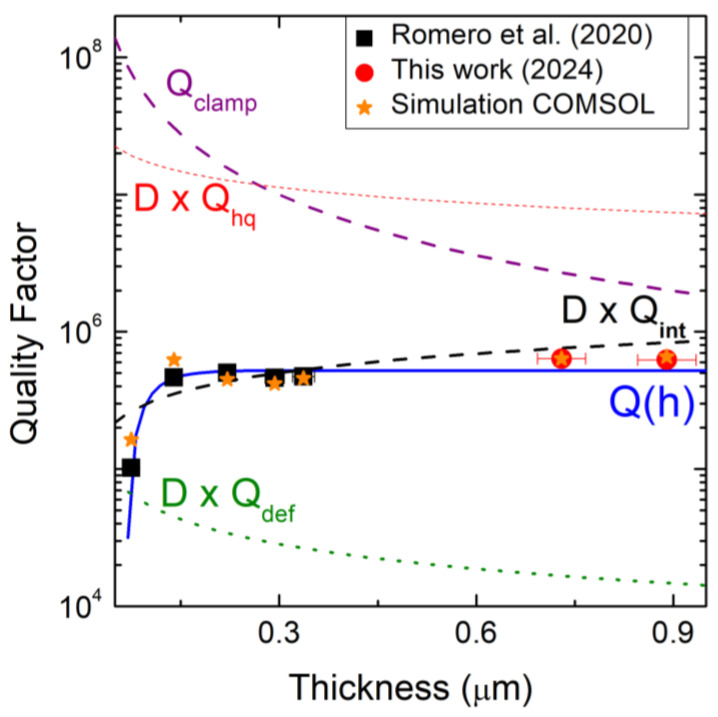
Mean values of the measured Q-factor with different thicknesses. Black squares refer to the total Q-factor of Romero et al.’s data [20]; red circles refer to our samples, while the dashed blue curve is the theoretical fit of the total quality factor. The dashed red and green curves are, respectively, the limit for the Q-factor when only the high-quality (or rich-defect) layer is considered in the resonator. The dashed purple curve is the external energy dissipation resulting from clamping loss. The dashed black curve is obtained when, for the total Q-factor, it is possible to neglect external energy dissipation via clamping loss, considering only intrinsic energy loss. The orange stars represent data simulated with COMSOL Multiphysics^®^.

In Figure 7, the black squares and red circles indicate the experimental values of the quality factor Q(h) determined during the first step of measurements, calculating the resonance frequency and FWHM ratio, with error bands found by error propagation. The blue line indicates the theoretical fit given by Equation (A1). The dashed red and green lines represent the $DxQ_{hq}$ and $DxQ_{def}$, respectively; the limit for the Q-factor when only the high-quality (or rich-defect) layer of the resonator is considered explains the huge difference (two orders of magnitude) between the two curves. The dashed purple curve is $Q_{clamp}$ calculated using the analytical expression (A4) in Appendix A for a beam resonator with mean stress σ(h). Neglecting the $Q_{clamp}$ curve, the $DxQ_{hq}$ curve is about two orders of magnitude greater than the total quality factor, and it is related to the possibility of fabricating beams in which only a high-quality film can be considered. In the literature, this result has been achieved for back-side-etched resonators. The lower $DxQ_{def}$ curve refers to the quality factor of a resonator in which the rich-defect layer has a thickness greater than that of the high-quality layer, so in this case, a worsening of the intrinsic dissipation occurs, and the quality factor decreases with the thickness. The dashed black line $DxQ_{int}$ shows how the contribution of clamping losses cannot be neglected in Equation (A1). Despite this, when it comes to highly stressed resonators, clamping loss is often neglected; the main contribution comes from the intrinsic dissipation. In this case, $DxQ_{int}$ is used, but from the graph in Figure 5, it is easy to observe the deviation of Q from $DxQ_{int}$, so the external clamping loss must be considered. Therefore, when the 3C-SiC film thickness increases, the highest quality factor is about $6.3\times 10^{5}$ at around 1 µm of thickness, and to achieve this, all the contributions must be considered—intrinsic and external. The energy-dissipation mechanisms at this thickness result from the intrinsic dissipation, considering mainly the volumic contributions for both high-quality and rich-defect layers in the bilayer system, and the external clamping losses related to the phonon tunneling from the beam to the substrate through the clamping points in the form of acoustic radiation. Even in the total Q-factor trend, there is a high congruence between our data and the results in the literature. Therefore, this model for quality factor developed by Romero et al. [20], which considers the two main energy-dissipation mechanisms (intrinsic and external), and the bilayer system model for the intrinsic contributions, can be used to determine the quality factor and the material properties of (111) 3C-SiC-undoped film on silicon substrate. Achieving this high-value quality factor, at 1 µm thickness, is a key result for application fields in which high resolution and sensitivity are fundamental. The experimental results obtained in this section have been used as a reference for the numerical modeling in the following section.

## 5. Numerical Simulation of Damping and Resonant Frequency Analysis

Damping defined by an isotropic loss factor is proportional to the displacement amplitude. Usually, the conversion between the damping ratio and loss factor damping is considered at resonant frequency, and then η≈2ζ where η is inversely proportional to the Q-factor:

(8) $$Q=\frac{1}{\eta }$$

One of the most obvious manifestations of damping is the amplitude decay during free vibrations. In a structural dynamics analysis, modeling the damping is important. The main reason to include damping in an eigenfrequency analysis is to estimate how much different resonances will be dampened. When using COMSOL Multiphysics, it is possible to include loss factor damping through the Damping subnode via a material model. The Damping node adds Rayleigh damping by default and the damping parameter ξ is expressed in terms of the mass m and the stiffness k as shown below.

(9) $$\xi =\alpha_{dM}m+\beta_{dK}k$$

Rayleigh damping is proportional to a linear combination of the stiffness and mass. In the following equation, loss information appears as a multiplier for the elastic constitutive matrix $D^{c}$

(10) $$D^{c}=\left(1+j\eta_{s}\right)D$$

**Figure 8:**
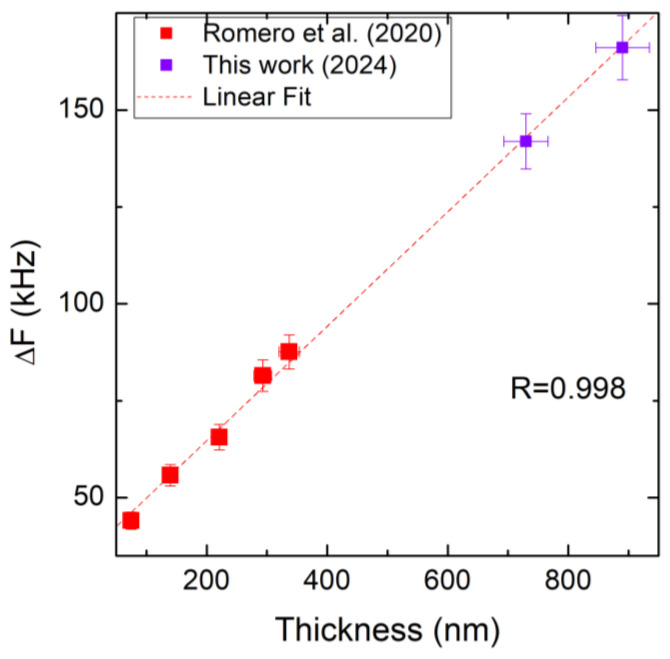
The difference between the values of the experimental and simulated frequencies as a function of the thickness, distinguishing Romero data [20] (red squares) and our data (violet squares).

The use of loss factor damping traditionally refers to a scalar-valued loss factor η_s_; it is a value deduced from true complex-valued material data. Comparing the experimental resonance frequency and the simulated data, we can identify some limitations of the current damping models, especially with respect to a double-clamped configuration. The simulated resonance frequency points are always greater than the experimental ones, and their difference is not constant. In Figure 8, we plot the difference between the values of the experimental and simulated resonance frequencies as a function of the thicknesses; there is a linear correlation (R = 0.998) between resonance frequency and thickness (*t*). The graphical points show an evident linear proportionality.

(11) ∆F=kt+c

This difference between the experiment and the numerical simulation is attributed to an effect of the damping on the resonance frequency that has not been perfectly described in the standard model reported in COMSOL.

**Figure 9:**
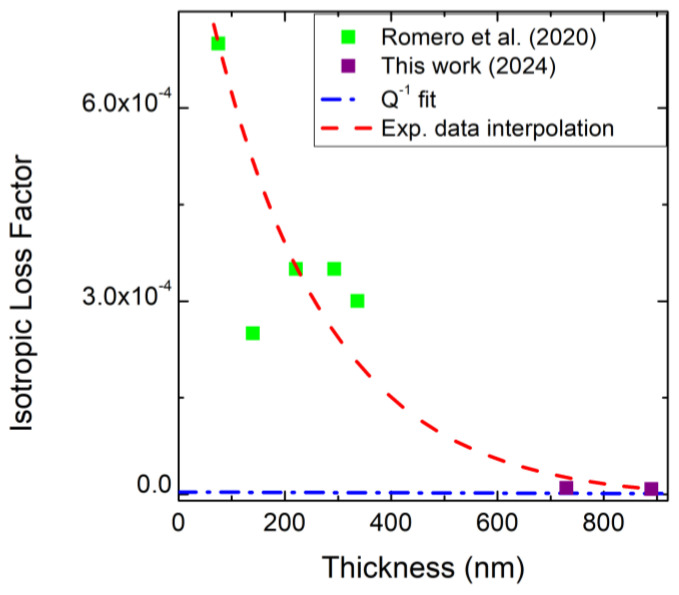
Relation between η vs. thickness: light green squares represent the isotropic loss factor of Romero et al.’s samples [20], while purple squares represent our data. The blue dashed–dotted curve is the $Q^{-1}$ fit. The red dashed curve represents experimental data interpolation.

Another aspect can be observed using these numerical simulations. As reported in Equation (8), the loss factor should be inversely proportional to the Q factor. If we look at the data reported in Figure 9, we can observe that only for the thicker layers do we observe this proportionality between the isotropic loss factor and the Q-factor. Instead, at reduced thickness, there is a large difference between the isotropic loss factor obtained by fitting the experimental data and the theoretical expected value. This is probably due to the large influence of the thinner films on the defects at the 3C-SiC/Si interface, which increase the isotropic loss factor of the material. For thicknesses higher than 700 nm, instead, the defects at the 3C-SiC/Si interface have a less pronounced effect on the mechanical properties of the resonator and the loss factor approach theoretical value depicted in Equation (8).

## 6. Conclusions

This work aims to verify if our data based on results from the literature confirm the results of the quality factor model presented by Romero et al. [20]. Combining all the equivalent 3C-SiC (111) dual-clamped beams with a low doping level and the same length, it is possible to compare seven different samples with thicknesses ranging from 0.075 to 0.89 µm. For all these wafers, the residual stress and dilution factor have been calculated, with analytic expressions, in addition to all the quality factor parameters distinguishing the two energy-dissipation mechanisms (intrinsic and external). In the intrinsic dissipation, the 3C-SiC films are associated with a bilayer system, in which there are two different layers: a rich-defect layer near the 3C-SiC/Si interface and a high-quality layer above the rich-defect layer, which has a lower defect density. The external dissipation mechanism occurs via clamping losses, determined by an analytic expression. Although in highly stressed film, the clamping loss is often neglected, in our case, we demonstrated how quality factors offer multiple contributions, both intrinsic and external. For all the samples, a quality factor with an average value of $5.5\times 10^{5}$ was measured and increasing the 3C-SiC film thickness resulted in the quality factor reaching $6.3\times 10^{5}$ in correspondence with 1 µm thickness of the 3C-SiC layer. This is quite a high value that allows us to achieve a better sensitivity and resolution in application fields. A high coherence between our data and the results in the literature has been observed, so the quality factor model developed by Romero et al. [20] represents an improvement for the study of the properties of SiC layers with high resolution.

COMSOL simulations show that, by introducing an isotropic loss factor, it is possible to obtain the experimental Q-factor in the entire range of thicknesses listed this paper. The resonance frequencies have not been obtained with accuracy using the isotropic loss factor, so this numerical model should be improved to consider these differences, which increase with the thickness of the 3C-SiC layer. Finally, it has been observed that the isotropic loss factor is high for thin 3C-SiC layers and approaches the theoretical value for 3C-SiC thicknesses above 700 nm. This numerical model can also be used to observe the importance of the defective layer at the 3C-SiC/Si interface with regard to the performance of the analyzed MEMSs.

## Figures and Tables

**Table 1:** Overview of Q-factor, residual stress and resonance frequency measurements on 3C-SiC samples.

Wafer Id	Si Type	Doping	Thickness (µm)	Res. Stress (MPa)	Res. Frequency (kHz)	Q-Factor
W1	<111>	NID	0.89	1010	312.20	624,400
W2	<111>	NID	0.73	982	318.07	636,150

Q-factor values refer to 1000 µm beam length.

## Data Availability

Data will be made available on request.

## Appendix A

According to the Q-factor model reported in [20] for 111 3C-SiC resonators, the overall Q factor of the devices can be expressed as follows:

(A1) $$Q^{-1}=D^{-1}Q_{int}^{-1}+Q_{clamp}^{-1}$$

assuming the thermoelastic damping contribution is negligible because of the reduced thickness of the layers. In Equation (A1), Q is the overall Q-factor of the resonator, $Q_{int}$, $Q_{clamp}$ are the intrinsic and clamp contributions to the Q-factor and *D* is a dilution factor given by

(A2) $$D^{-1}\left(h\right)\approx \left(2\lambda +\pi^{2}\lambda^{2}\right)$$

with

(A3) $$\lambda =\frac{h}{L}\sqrt{\frac{E}{12\sigma \left(h\right)}}$$

where E is SiC’s Young’s modulus, h is the thickness of the SiC layer, L is the length of the beam resonator and σ(h) is the average residual stress of the layer, depending on its thickness.

The clamp contribution to the Q-factor, due to the emission of acoustic phonons towards the bulk of the device during vibration, can be expressed as follows:

(A4) $$Q_{clamp}=\;\alpha \;\frac{3\rho_{s}}{2\rho }\sqrt{\frac{E_{s\;}\rho }{2\;\sigma \left(h\right)\rho_{s}}}\;\frac{L}{h}$$

in which $E_{S}$ is silicon’s Young’s modulus, ρ, $\rho_{S}$ are the densities of SiC and Si, respectively, and α is a fitting parameter that is kept constant during the fitting procedure.

The intrinsic contribution to the Q-factor, on the other hand, can be calculated as follows:

(A5) $$Q_{int}^{-1}\left(h\right)=\;\left(\frac{h-h_{0}}{h}\right)\left\{{\left[Q_{hq}^{surf}\left(h\right)\right]}^{-1}+{\left[Q_{hq}^{vol}\left(h\right)\right]}^{-1}\right\}+\left(\frac{h_{0}}{h}\right)\left\{{\left[Q_{def}^{surf}\right]}^{-1}+{\left[Q_{def}^{vol}\right]}^{-1}\right\}$$

where $h_{0}$ is the thickness of the defective SiC layer, whereas $Q_{hq}^{surf}$, $Q_{hq}^{vol}$, $Q_{def}^{surf}$, $Q_{def}^{vol}$ are the surface and volume’s contributions to the intrinsic Q-factor, respectively, due to the defective ($h_{0}$-thick) and high-quality SiC layers used to create the resonators.

The surface contributions to the intrinsic Q-factor can, in turn, be expressed as functions of the layer thickness h in the following way:

(A6) $$Q_{hq}^{surf}=\beta_{hq}\left(h-h_{0}\right)$$

and

(A7) $$Q_{def}^{surf}=\beta_{def}\left(h-h_{0}\right)$$

As can be seen from Equations (A1)–(A7), the overall Q-factor model proposed in [20] contains six fitting parameters, which are $Q_{hq}^{vol}$, $Q_{def}^{vol}$, $\beta_{hq}$, $\beta_{def}$, $h_{0}$, and α. These parameters can be determined in principle by a best-fitting procedure in which the model is compared with experimental Q-factor data possibly measured on resonators with different thicknesses (h).
